# Functional connectivity of dorsolateral prefrontal cortex predicts cocaine relapse: implications for neuromodulation treatment

**DOI:** 10.1093/braincomms/fcab120

**Published:** 2021-06-02

**Authors:** Tianye Zhai, Betty Jo Salmeron, Hong Gu, Bryon Adinoff, Elliot A Stein, Yihong Yang

**Affiliations:** 1 Neuroimaging Research Branch, Intramural Research Program, National Institute on Drug Abuse, National Institutes of Health, Baltimore, MD 21224, USA; 2 Veterans Affairs North Texas Health Care System, Dallas, TX 75216, USA; 3 Department of Psychiatry, University of Texas Southwestern Medical Center, Dallas, TX 75390, USA; 4 Department of Psychiatry-Residency, School of Medicine, University of Colorado, Aurora, CO 80045, USA

**Keywords:** dlPFC, cocaine relapse, functional connectivity, prediction, treatment outcome

## Abstract

Relapse is one of the most perplexing problems of addiction. The dorsolateral prefrontal cortex is crucially involved in numerous cognitive and affective processes that are implicated in the phenotypes of both substance use disorders and other neuropsychiatric diseases and has become the principal site to deliver transcranial magnetic stimulation for their treatment. However, the dorsolateral prefrontal cortex is an anatomically large and functionally heterogeneous region, and the specific dorsolateral prefrontal cortex locus and dorsolateral prefrontal cortex-based functional circuits that contribute to drug relapse and/or treatment outcome remain unknown. We systematically investigated the relationship of cocaine relapse with functional circuits from 98 dorsolateral prefrontal cortex regions-of-interest defined by evenly sampling the entire surface of bilateral dorsolateral prefrontal cortex in a cohort of cocaine dependent patients (*n* = 43, 5 Fr) following a psychosocial treatment intervention. Cox regression models were utilized to predict relapse likelihood based on dorsolateral prefrontal cortex functional connectivity strength. Functional connectivity from only 3 of the 98 dorsolateral prefrontal cortex loci, one in the left and two in the right hemisphere, significantly predicted cocaine relapse with an accuracy of 83.9%, 84.6% and 85.4%, respectively. Combining all three loci significantly improved prediction validity to 87.5%. Protective and risk circuits related to these dorsolateral prefrontal cortex loci were identified that have previously been implicated to support ‘bottom up’ drive to use drug and ‘top down’ control over behaviour together with social emotional, learning and memory processing. Three dorsolateral prefrontal cortex-centric circuits were identified that predict relapse to cocaine use with high accuracy. These functionally distinct dorsolateral prefrontal cortex-based circuits provide insights into the multiple roles played by the dorsolateral prefrontal cortex in cognitive and affective functioning that affects treatment outcome. The identified dorsolateral prefrontal cortex loci may serve as potential neuromodulation targets to be tested in subsequent clinical studies for addiction treatment and as clinically relevant biomarkers of its efficacy. Zhai et al. identify three dorsolateral prefrontal cortex (dlPFC)-centric circuits that predict cocaine relapse with high accuracy, providing insights into the multiple roles of the dlPFC in brain functioning that affects treatment outcome and suggesting the dlPFC loci as potential neuromodulation targets for addiction treatment.

## Graphical Abstract

**Graphical Abstract:**
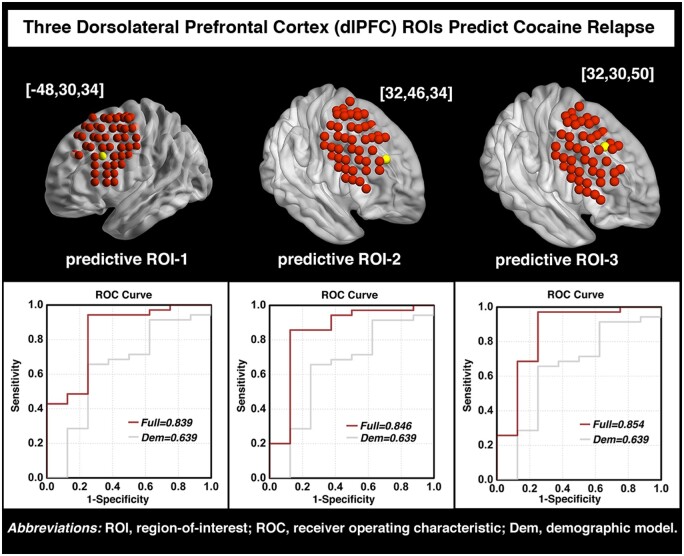

## Introduction

Drug addiction has serious negative impact on the individual, family, community and society at large, resulting in hundreds of billions of dollars in direct and indirect public costs annually attributed to crime, health care and loss of productivity.^1^^,^^2^ Pre-clinical and human research supports that drug addiction should be considered and treated as an acquired, highly relapsing, chronic brain disease.^1^^,^^3^^,^^4^ Unfortunately, current addiction treatments remain relatively ineffective, with relapse rates post-treatment about 70% and are further compounded by high dropout rates (∼35%) before treatment completion, even for treatment-seeking patients.^4^

These data reflect the core feature of the addiction phenotype, i.e., loss of control over drug use, which has been attributed to abnormalities in multiple cognitive and affective domains, including decision-making,^5^ inhibitory control,^6^ craving,^7^ memories function^8^ and regulation of negative emotional.^9^ These abnormalities are closely associated with dysfunctions in top-down executive control, which is mediated, at least in part, by regions within the dorsolateral prefrontal cortex (dlPFC) and their ‘downstream’ functional circuits.^10^^,^^11^ As the core of the Executive Control Network (ECN),^12^^,^^13^ the dlPFC is involved in both cognitive and affective domains that are impaired in substance use disorders.

For example, in the cognitive domain, the dlPFC plays a pivotal role in intertemporal value-based decision-making in computing the value of long-term consequences of delayed vs. more immediate rewards,^14^ which is significantly dysregulated in substance use disorders (SUDs),^15^ where SUD patients have a propensity for choosing more immediate smaller rewards over larger, delayed rewards when assessed via temporal discounting behavioural choice paradigms.^5^^,^^16^ The dlPFC is also implicated in risk avoidance. Attenuated activity is observed in the right dlPFC that is accompanied by deficits in risk avoidance in methamphetamine dependent patients during the Balloon Analogue Risk Task.^17^ The dlPFC is well documented to be involved in inhibitory control,^18–21^ and working memory,^22–24^ which have been consistently demonstrated to show impairment in SUD.^3^^,^^6^^,^^10^^,^^25^^,^^26^

In the affective domain, SUD patients often suffer from a persistent negative emotional state similar to that seen in depressive disorders, which also show high comorbidity with SUD.^27^^,^^28^ While the dlPFC is consistently shown to be hypoactive in major depressive disorder,^29^ increased activity in this brain area is associated with recovery/remission of depressive symptoms.^30–32^ Lesion studies causally link damage to the bilateral dlPFC with higher levels of depressive symptoms.^31^ Moreover, the dlPFC is also implicated in explicit and implicit emotion regulation,^33^ which would be expected to be especially taxed during the preoccupation phase of addiction.^3^

In light of the overarching role of the dlPFC in multiple cognitive and affective domains that are also dysregulated in SUD, it has been hypothesized that modulation of this region may help produce alterations in brain circuits relevant to drug seeking and taking behaviour. For example, in healthy participants, real-time neurofeedback of functional MRI (fMRI) signals derived from the dlPFC can enhance vigilance and working memory task performance.^34^^,^^35^ Inhibitory transcranial magnetic stimulation (TMS) targeting the left dlPFC increases choice for immediate versus delayed rewards.^36^ Clinical studies using excitatory high frequency repetitive TMS (rTMS) also targeting the left dlPFC, reduces craving in cocaine and nicotine dependent populations.^37–39^ More recently, an open label pilot study showed promising results for high frequency rTMS delivered to left dlPFC in reducing relapse to cocaine use compared to a standard pharmacological treatment.^40^

Choosing the optimal stimulation location in neuromodulation may be the most crucial determinant for its treatment effectiveness. Investigators and addiction treatment clinicians have utilized various dlPFC TMS loci, e.g. F3 from the 10–20 EEG system,^41^ or various coordinates with the aid of a neuronavigation system.^37^^,^^40^ Unfortunately, although multiple regions-of-interest (ROIs) within the dlPFC have been consistently implicated in brain functions related to SUD, the exact dlPFC loci and their associated functional circuits that underlie drug relapse remain unknown, leaving the selection of any location usually lacking a compelling justification that is most often simply following a previously published study. The parameter space (e.g. location, pulse width, intensity, frequency, duration, etc.) to determine optimal efficacious neuromodulation target is huge to the point of being virtually impossible to explore in human clinical studies in a systematic, comprehensive manner. It is, however, possible to characterize functional connectivity from various dlPFC loci in cocaine dependent individuals following treatment completion (traditional treatment such as psychosocial treatment) to identify dlPFC locus and its functional circuits potentially related to relapse, which might subsequently be prioritized in developing new TMS treatment targets.

**Figure 1:**
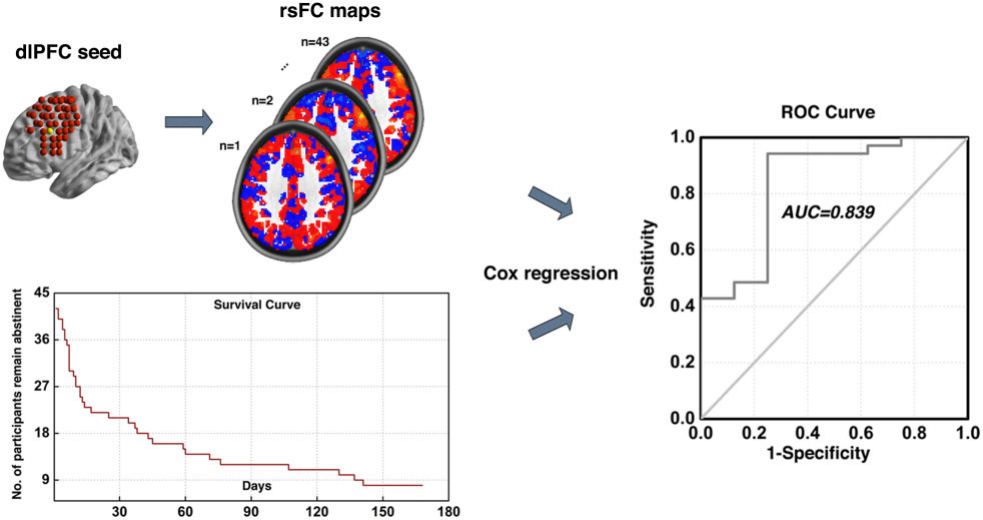
**Illustration of analytical procedure. Illustration of our analytical procedure using one dlPFC locus as exemplar.** First, the whole brain functional connectivity maps for each subject was calculated using the example dlPFC locus as seed. By combining these functional connectivity maps with the post-treatment information obtained during the follow-up period, we utilized the Cox regression based modelling to predict cocaine relapse. This procedure was conducted recursively for all 98 dlPFC sub-regions covering the entire bilateral dlPFC area.

As such, we interrogated the functional circuitry from multiple surface-level ROIs covering the entire dlPFC and their relationship to post-treatment relapse. We sought to pursuit better understanding of dlPFC loci and their associated functional circuits that underlie cocaine relapse, and provide potential neuromodulation targets to be tested in future clinical studies for addiction treatment. Specifically, we utilized the Cox regression model combined with resting-state functional connectivity to produce a comprehensive map of dlPFC related circuits in a cohort of cocaine dependent individuals who were imaged after completing an inpatient psychosocial treatment regimen and subsequently followed post-treatment for 24 weeks (Fig. 1). We aimed to identify loci within the large and heterogeneous brain region of dlPFC that relate to the likelihood of relapse to cocaine use.

## Materials and methods

### Participants

Forty-five participants who completed treatment and follow-up from local residential treatment programs are included in the current analysis. Study inclusion criteria included right-handedness, meeting criteria for cocaine dependence (DSM-IV), no history of major medical illness, estimated IQ over 70 (limited cognitive resources are engaged during resting-state fMRI acquisition) based on the Wechsler Test of Adult Reading, not meeting criteria for any neurological or active Axis I disorder (other than substance dependence), and not on any psychotropic medications. The study was reviewed and approved by the Institutional Review Boards of the University of Texas Southwestern Medical Center and the Veterans Administration North Texas Health Care System. Written informed consent was obtained from each participant after the nature and possible consequences of the study were explained. The current analysis is using data from a treatment-seeking cocaine dependent cohort that have been previously reported.^42–46^

### Treatment and assessment procedures

Cocaine dependent participants were admitted to one of three treatment programs as soon as possible after their last reported use of cocaine. All three treatment programs utilized the Minnesota Model Psychosocial treatment approach.^47^ Demographic information and drug use history were collected during the first week of treatment. MRI scans were conducted during the final week of inpatient treatment. Urine drug screens (UDS) were conducted to verify abstinence. Several bench tasks/psychological measurements were also assessed including a Barrett Impulsivity Scale (BIS-11a) which was measured during the first/second week of the treatment; a Cocaine Craving Questionnaire (CCQ-Brief), a Conners’ Continuous Performance Test II (CPT), a Wisconsin Card Sorting Task (WCST) and an Iowa Gambling Task (IGT) which were conducted during the same week of participants’ MRI scan visit; see previous publications for more details.^42^^,^^43^^,^^45^

Following discharge, participants were followed for up to 24 weeks or until relapse to stimulant use, whichever came first. During this period, two follow-up sessions occurred each week with one session over the phone and the other in-person. A structured interview assessing substance use since the previous visit (or since discharge from the treatment programs for the first visit) and a UDS (for the in-person session) were completed. Relapse was defined as any use of cocaine or amphetamine (either by self-report or by UDS) post-discharge or missing two consecutive appointments without contact.^48^^,^^49^ Date of relapse was recorded as the day of drug use or the day of the first missed appointment if lost to follow-up. Participants who failed to maintain abstinence were discharged from the study and excluded from further follow-up contact.

### MRI acquisition

MRI scans were obtained using a Philips 3 T scanner with an eight-channel radio-frequency coil (Philips Medical Systems, Best, The Netherlands). For each participant, six minutes of whole brain blood oxygen level dependent (BOLD) resting-state fMRI data were acquired in the axial plane parallel to the AC-PC line using a single-shot, echo-planar imaging sequence (echo time = 25ms, repetition time = 1.7s, flip angle = 70°, spatial resolution = 3.25 mm × 3.25 mm × 3 mm with no gap). Participants were instructed to keep their heads still and eyes open during the resting-state scan. A high-resolution anatomical T1-weighted image was also acquired from each participant using a 3D magnetization-prepared rapid gradient-echo sequence (echo time = 3.8 ms, repetition time = 8.2 ms, flip angle = 12°, spatial resolution = 1mm × 1 mm × 1 mm).

### Data processing pipeline

The data processing pipeline consisted of six conceptual steps: (1) image preprocessing; (2) dlPFC functional connectivity calculation; (3) voxel-wise Cox regression analysis; (4) thresholding and generating composite indices; (5) Cox model fitting for brain–behaviour relationship using the composite indices and model evaluation (ROC analysis); and (6) cross validation. Steps 3 to 6 were adapted and modified from the pipeline proposed by Shen et al.,^50^ which provided a framework for modelling individual behaviours with whole brain functional connectivity. Supplementary Fig. 1 shows the schematic diagram of our analyses pipeline using one dlPFC locus as an exemplar.

### Image pre-processing

Imaging data preprocessing and functional connectivity analyses were conducted using AFNI (v17.0.06, http://afni.nimh.nih.gov/afni/) and SPM12 (http://www.fil.ion.ucl.ac.uk/spm/software/spm12/) software packages. The Cox regression analysis was conducted using a customized program on the Matlab platform (R2017a, The MathWorks, Inc., Natick, Massachusetts). Data preprocessing included: discarding the first five volumes to allow the magnetic resonance signal to reach steady state, slice timing correction (*3dTshift, AFNI*), volume registration (*3dvolreg, AFNI*), detrending (up to the 3rd order, *3dDetrend, AFNI*) and head motion correction (*3dTproject, AFNI*). Head motion was also evaluated at the frame-by-frame level to further control image quality by using pair-wise displacement calculated based on the Euclidean distance (*1d_tool.py, AFNI*). Volumes with displacement > 0.35 mm were censored, participants were excluded if their mean head motion across volumes were greater than 0.2 mm or their percentage of censored volumes exceeding 20%. Two participants were excluded due to head motion exceeding this threshold, leaving 43 participants in the final analyses. Signal from white matter and cerebrospinal fluid were regressed out as a marker of non-neuronal noise (*3dTproject, AFNI*). A band-pass filter was applied to select low-frequency fluctuations between 0.012 Hz and 0.1 Hz (*3dTproject, AFNI*).^51^ The blood-oxygen-level-dependent (BOLD) data were normalized to standard Montreal Neurological Institute image space and resampled to 2 mm isotropic resolution (SPM12).

### Dorsolateral prefrontal cortex functional connectivity

As the dlPFC is a large and heterogeneous region, we performed a comprehensive and systematic analysis of the entire dlPFC (both left and right hemispheres). We first defined the dlPFC borders based on the probabilistic ‘Harvard-Oxford cortical and subcortical structural atlases’ (3rd sub-layer, which covers the middle frontal gyrus areas that partly overlap with the BA9 and BA46) provided within FSL (v5.0.9, https://fsl.fmrib.ox.ac.uk/fsl/fslwiki). In consideration of both computational efficiency and the stimulation focality of TMS, for which we intend to provide guidance with our results, we down-sampled this bilateral dlPFC mask to 8 mm isotropic resolution and selected ROIs near the surface of the cortex that are potentially accessible directly by TMS. This yielded 98 sets of coordinates around which we created 98 4 mm radius spherical seed ROIs that evenly sampled the surface level of the bilateral dlPFC. Cross-correlation coefficient (CC) maps of each participant were generated by correlating the time course of each of the 98 seeds with that of each voxel in the whole brain. Fisher’s Z-transformation was applied to the CC maps resulting in z maps that were used in a subsequent voxel-wise Cox regression. All subsequent analyses were conducted within voxels in a grey matter constrained probabilistic mask.

### Voxel-wise Cox regression analysis

To investigate the relationship between dlPFC functional connectivity and cocaine relapse, we utilized the Cox regression model to perform a voxel-wise whole brain search for dlPFC-based circuits that predicted cocaine relapse, with the factors of age, sex, years of education, daily cigarette use and head movement (FD) during scanning as covariates. The beta coefficient (weighting of the model fit) of each voxel (i.e. its connectivity with that dlPFC seed voxel) was estimated via Cox regression. We then obtained the relative hazard ratio (HR) values by calculating the exponential of the beta coefficient values to generate HR maps of all participants.

### Thresholding and composite index generation

All HR maps from the voxel-wise Cox regression were thresholded at *P *<* *0.001 (for both positive and negative beta coefficients). These HR maps were used to generate: (I) a set of ‘protective’ circuits, which are comprised of voxels with HR value less than 1 (or negative beta coefficient, indicating less likelihood of relapse within the follow-up period with stronger functional connectivity); and a set of ‘risk circuits’, comprised of voxels with HR value greater than 1 (or positive beta coefficient, indicating higher likelihood of relapse within the follow-up period with stronger functional connectivity); and (ii) two composite indices: protective (indexP) and risk (indexR) indices using linear summation of all voxels within the ‘protective’ and the ‘risk’ circuits, respectively, for each participant.

### Brain–behaviour relationship (model fitting, ROC analysis and model comparison)

We fit both indexP and indexR into the final Cox model, with age, gender, years of education, daily cigarette use and head movement during scanning as covariates. A receiver operating characteristic (ROC) analysis was conducted to determine the predictive power of the model. The ROC curve was generated by calculating the sensitivity and specificity at multiple thresholds, then the area under curve (AUC) was obtained for the ROC curve and used as a measure of prediction accuracy. We used the log-likelihood tests for comparison between different models.

### Cross validation and permutation test

To validate the relapse prediction models and to test their potential in predicting new individuals, we conducted the above analyses from steps 3 to 5 in a leave-one-out (LOO) manner, i.e., we repeated the whole analysis 43 times, excluding one participant each time and using the remaining 42 participants to estimate indices or intermediate results of this individual participant. After all LOO steps were finished, we stacked the HR maps of all 43 LOO steps, binarized and thresholded at 85% to generate the group level heat map, identifying a set of risk circuits and a set of protective circuits from each dlPFC seed that uniquely contributed to cocaine relapse. Three dimensional visualization of these circuits was implemented using the BrainNet Viewer tool.^52^

Permutation testing was performed to empirically determine significance thresholds and thus to control for overfitting. We repeated the entire analysis 10 000 times, each time with the predictor (indices based on dlPFC functional connectivity) and outcome (days till relapse) pairs randomly permuted to generate our data/model specific empirical *null* distribution. The *P*-value of the AUC was then derived based on the ranking of the actual AUC value in this empirical *null* distribution. For our 98 dlPFC ROIs, the AUC values were considered statistically significant if the *P*-values were <5.1 × 10^−4^ (0.05/98 for multiple comparison correction).

### Data availability

Raw data generated in the current study contain personally identifiable information that could compromise the privacy of research participants if shared publicly. Derived data supporting the findings of this study are available from the corresponding author contingency of institutional approval, upon reasonable request.

## Results

### Demographic and clinical characterization

Of the 45 participants, 43 remained in the final analysis following removal of 2 participants with excessive head motion. This cohort included 5 females and 38 males, with a mean (SD) age of 43.4 (7.2), years of education of 12.5 (2.1), years of cocaine use of 8.3 (5.2), and cigarette per day of 11.4 (10.4). As shown in the survival curve in Fig. 1 (lower left corner), the number of participants who remained abstinent dropped rapidly during the first 30 days, and by the end of the 168-day follow-up period, 35 out of 43 participants had relapsed.

### Heterogeneity in predicting relapse across multiple dlPFC ROIs

**Figure 2:**
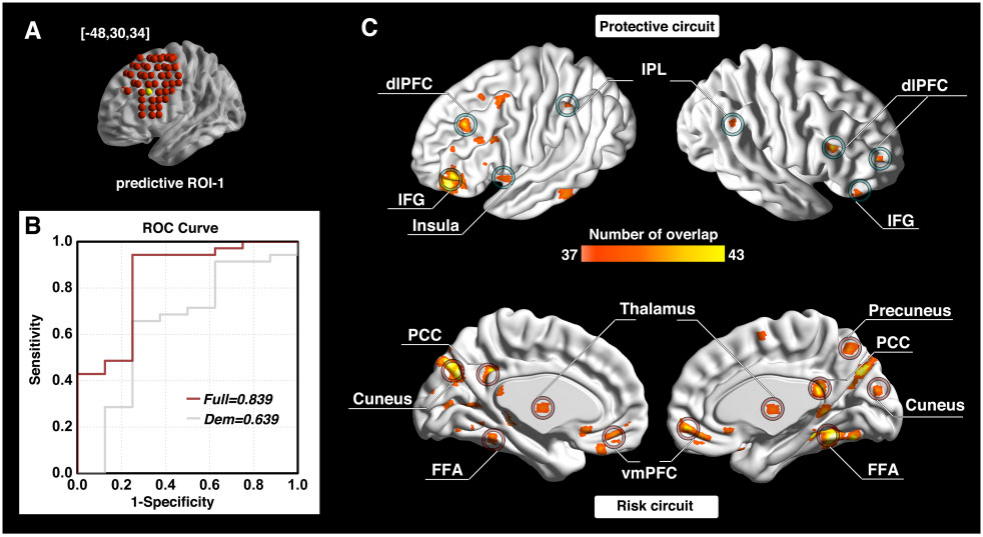
**Prediction accuracy and functional circuits that contribute to cocaine relapse prediction (predictive ROI-1).** Relapse prediction based on predictive ROI-1 [−48, 30, 34] (yellow ball in **A**) yielded AUC value of 0.839 (**B**). Two functional circuits were identified that contributed to cocaine relapse: The protective circuit includes bilateral dlPFC, inferior frontal gyrus (IFG), inferior parietal lobule (IPL), and the left insula (**C**, upper); the risk circuit includes the ventromedial prefrontal cortex (vmPFC), thalamus and the posterior cingulate cortex (PCC)/precuneus (default mode network regions), as well as cortical regions, including visual and motor regions (**C**, lower). Dem = demographic model (prediction model only use demographic information); dlPFC = dorsolateral prefrontal cortex; FFA = fusiform face area; IFG = inferior frontal gyrus; IPL = inferior parietal lobule; PCC = posterior cingulate cortex; ROC = receiver operating characteristic; ROI = region of interest; vmPFC = ventromedial prefrontal cortex.

**Figure 3:**
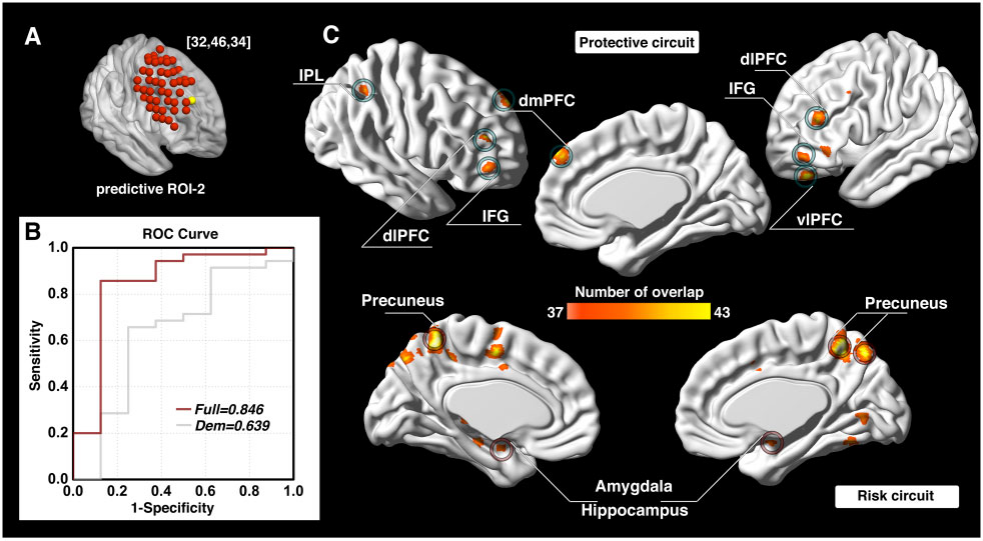
**Prediction accuracy and functional circuits that contribute to cocaine relapse prediction (predictive ROI-2).** Relapse prediction based on predictive ROI-2 [32, 46, 34] (yellow ball in **A**) yielded AUC value of 0.846 (**B**). Two functional circuits contributed to cocaine relapse: The protective circuit mainly includes bilateral dlPFC, IFG, right IPL, dmPFC and left vlPFC (**C**, upper); the risk circuit mainly includes bilateral precuneus and amygdala/hippocampus (**C**, lower). Dem = demographic model (prediction model only use demographic information); dlPFC = dorsolateral prefrontal cortex; dmPFC = dorsomedial prefrontal cortex; IFG = inferior frontal gyrus; IPL = inferior parietal lobule; ROC = receiver operating characteristic; ROI = region of interest; vlPFC = ventrolateral prefrontal cortex.

**Figure 4:**
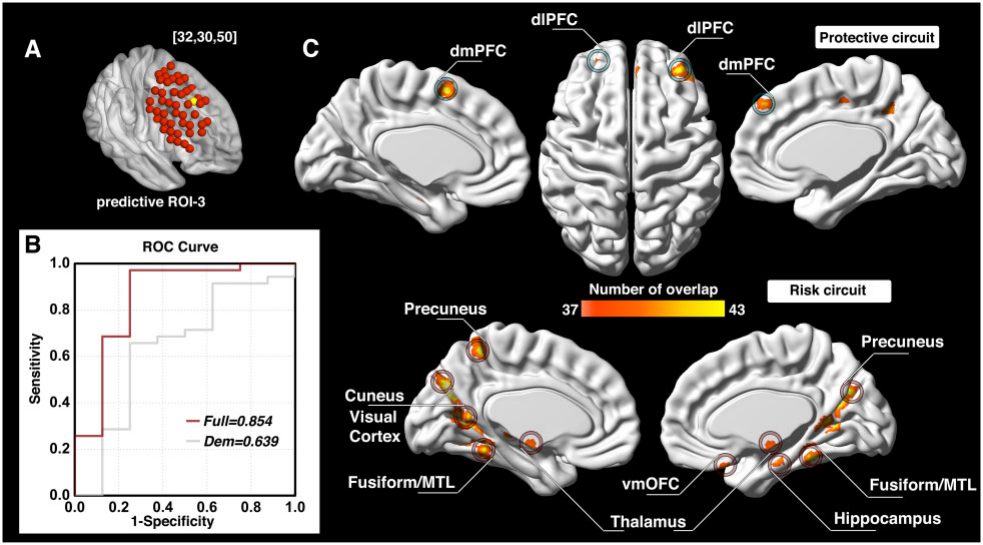
**Prediction accuracy and functional circuits that contribute to cocaine relapse prediction (predictive ROI-3).** Relapse prediction based on predictive ROI-3 [32, 30, 50] (yellow ball in **A**) yielded AUC value of 0.854 (**B**). Two functional circuits contributed to cocaine relapse: the protective circuit mainly includes bilateral dlPFC and dmPFC (**C**, upper); the risk circuit included the bilateral cuneus/visual cortex, vmOFC, thalamus, fusiform gyrus and the left precuneus (**C**, lower). Dem = demographic model (prediction model only use demographic information); dlPFC = dorsolateral prefrontal cortex; dmPFC = dorsomedial prefrontal cortex; MTL = medial temporal lobe; ROC = receiver operating characteristic; ROI = region of interest; vmOFC = ventromedial orbitofrontal cortex.

The investigation across the 98 ROIs covering the surface area of both the left and right dlPFC yielded prediction models with AUC values of ROC curves ranging from 0.343 to 0.854. Followed by a conservative correction (Bonferroni) for multiple comparisons, three of the 98 dlPFC ROIs (one on the left and two on the right side) significantly predicted cocaine relapse with their corresponding functional circuits (*P *<* *5.1 × 10^−4^, 0.05/98). The ROI on the left dlPFC (ROC curve showing AUC of 0.839) was located at Montreal Neurological Institute coordinates [−48, 30, 34] (Fig. 2A and B). We define this locus as ‘predictive ROI-1’ hereafter. Two ROIs on the right side that showed significant prediction accuracy (ROC curves showing AUC value of 0.846 and 0.854) were located at Montreal Neurological Institute coordinates [32, 46, 34] and [32, 30, 50] (Figs 3A and B and 4A and B). We define these two ROIs as ‘predictive ROI-2’ and ‘predictive ROI-3’, respectively.

### Neural circuits related to the three predictive ROIs confer protection against and risk for cocaine relapse

For each of the predictive loci, the nature of the Cox regression based survival analysis allowed us to identify two sets of dlPFC functional circuits that uniquely contributed to cocaine relapse: a set of ‘protective’ circuits, which consisted of voxels whose connectivity with the dlPFC ROI correlated negatively with relapse likelihood (the stronger functional connectivity, the less probability of relapse within the follow-up period); and a set of ‘risk’ circuits that consisted of voxels whose connectivity with the dlPFC ROI correlated positively with relapse likelihood (the stronger functional connectivity, the higher probability of relapse within the follow-up period). For predictive ROI-1 (Fig. 2C), the protective circuits comprised components of the canonical ECN,^53^ including the bilateral dlPFC, inferior parietal lobule (IPL), and inferior frontal gyrus (IFG); while the risk circuits comprised dlPFC connections to the canonical default mode network (DMN),^53^ including the bilateral ventromedial prefrontal cortex (vmPFC), posterior cingulate cortex (PCC)/precuneus and thalamus, along with visual and motor cortices, and the fusiform face area. For predictive ROI-2 (Fig. 3C), the protective circuits include the bilateral dlPFC, IFG, right IPL, dmPFC and left ventrolateral prefrontal cortex (vlPFC), while the risk circuits comprised primarily the bilateral precuneus and amygdala/hippocampus. For predictive ROI-3 (Fig. 4C), the protective circuits included the bilateral dlPFC and dmPFC, while the risk circuits included the bilateral cuneus/visual cortex, ventromedial orbitofrontal cortex (vmOFC), thalamus, fusiform gyrus, and left precuneus (see Supplementary Table 1 for a detailed list of circuit regions).

### Prediction model combining circuits from all three predictive ROIs

**Figure 5:**
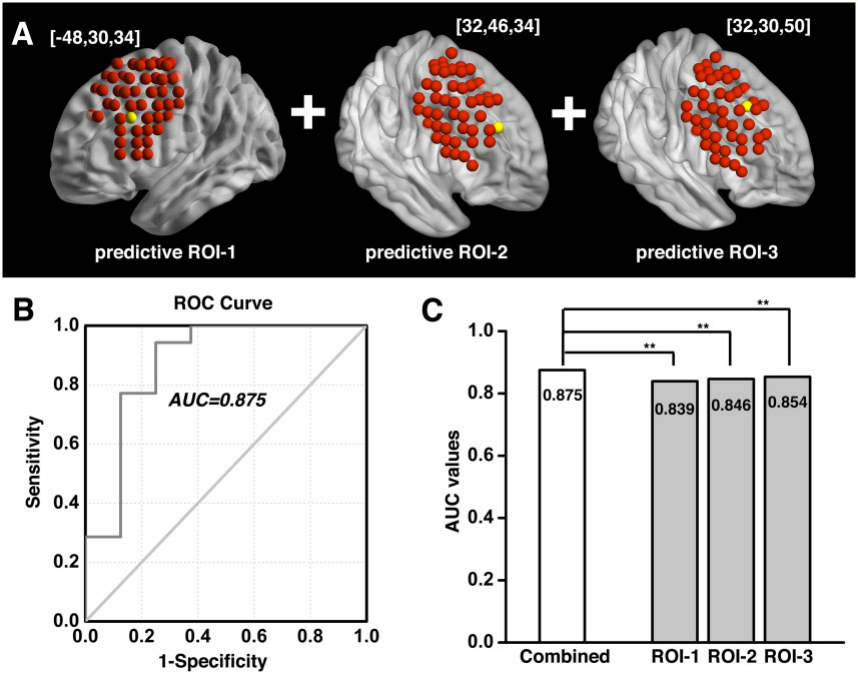
**Combined prediction model based on predictive ROI-1, -2 and -3.** When combining the protective and risk circuits from all three predictive ROIs (**A**), the prediction model yielded an AUC of 0.875 (**B**), showing significant improvement over each of the three individual predictive ROIs taken alone, based on log-likelihood testing (**C**). There is one *P*-value for the log-likelihood testing between models in each cross validation iteration, the mean of the these *P*-values was used to indicate the significance of the test, ** denotes *P* < 0.001. AUC = area-under-curve; ROC =receiver operating characteristic; ROI = region of interest.

To determine if prediction models from these three dlPFC loci explained variance in relapse in an overlapped or independent fashion, we combined the indices for the protective and the risk circuits from all three loci and built a *combined model*. As shown in Fig. 5A and B, the *combined model* (predictive ROI-1+ROI-2+ROI-3) yielded an AUC of 0.875, demonstrating a significant improvement over the predictive ability of each of the three predictive ROIs alone, based on log-likelihood testing (Fig. 5C, *P *<* *0.001 for all three comparisons).

### Control analyses and negative results

For a negative control, we tested our prediction modelling using six different sensory/motor seed ROIs that have been served as control sites previously.^54^ None of these control ROIs significantly predicted cocaine relapse; see Supplementary Table 2 for details. We also tested the relationship of the risk- and protective-circuits of the three predictive ROIs that we identified with several behavioural/psychological measurements in subsets that completed specific tests/questionnaires of our relapse prediction cohort. Specifically, the omission error, commission error, and reaction time from a continuous performance task (CPT, *n* = 40); preservation response from a Wisconsin Card Sorting Task (WCST, *n* = 37); total score on economical choices from an Iowa Gambling Task (IGT, *n* = 38); subjective measurement of the Barratt Impulsiveness Scale (BIS-11a, *n* = 42); and subjective measurement of the Cocaine Craving Questionnaire-Brief (CCQ-Brief, *n* = 42) were tested. None of these measurements significantly correlated with the functional circuits we identified that predict cocaine relapse; see Supplementary Table 3 for details. We further tested whether these behavioural/psychological measurements could predict cocaine relapse by utilizing the same prediction modelling as we used in the imaging-relapse analysis on these behavioural/psychological measurements, but none of these measurements significantly predicted cocaine relapse; see Supplementary Table 4 for details.

## Discussion

Using resting-state functional connectivity and a Cox regression-based prediction model, we systematically investigated the relationship between functional connectivity from multiple dlPFC ROIs and treatment outcome in a cohort of cocaine dependent individuals who had completed an inpatient, psychosocial treatment intervention. From the 98 ROIs covering the entire bilateral dlPFC surface area, we identified three ROIs (predictive ROI-1, 2 and 3) that demonstrated predictive validity of cocaine relapse within the 24-week study follow-up period. Moreover, the Cox regression allowed us to identify two sets of functional circuits for each of these three dlPFC ROIs, one related to risk for and the other related to protection against relapse (referred to as ‘risk circuit’ and ‘protective circuit’ hereafter). These circuits have previously been associated with dlPFC functions that are consistent with the cocaine dependence phenotype, as discussed below.

### The protective and risk circuits from predictive ROI-1

The protective circuit identified from the left hemisphere dlPFC ROI (predictive ROI-1; Fig. 2A) mainly include the bilateral dlPFC, IPL, IFG and the left insula. The dlPFC, IPL and IFG are major components of the canonical ECN, which has consistently been implicated in top-down executive control, response inhibition and performance of attentionally demanding cognitive tasks.^55–57^ Reduced functional connectivity strength within ECN regions has previously been reported in cocaine dependent individuals.^45^^,^^58^ This identified protective circuit has also been termed the ‘δ-network’ within a neuroeconomic based decision-making context, and serves as a ‘control’ network that takes long-term considerations into account when facing alternative valuation choices.^5^^,^^14^ Consistent with these previous findings, this identified protective circuit would suggest a stronger ‘control’ network lowers the likelihood of relapse (i.e. increase abstinence likelihood). Indeed, one of the key symptoms of SUD, impaired response inhibition, has been associated with abnormalities (generally reduced task-induced activation compared to healthy control individuals) in these same ECN regions (i.e. dlPFC, IFG and IPL).^56^^,^^57^^,^^59^ Longitudinal studies further show that impaired response inhibition, along with its underlying neural correlates, is associated with not only the onset of substance use in adolescents with little previous substance use experience, but also relapse in SUD individuals attempting to quit.^57^^,^^60^ The insula-centred Salience Network has been proposed to play a critical role in assigning cognitive resources based upon the saliency of homeostatically relevant external versus internal oriented stimuli via its interaction with the ECN and the DMN^61^; a disruption in such a role might underlie several major psychiatric and neurological disorders, including addiction.^55^^,^^62^^,^^63^ The protective dlPFC-insula connectivity found herein is reflective of the interaction between the ECN and the Salience Network. Individuals with higher connectivity strength in the circuit would have less likelihood of cocaine relapse, which could be attributed to the allocation of attentional and cognitive resources to more external oriented stimuli.

In contrast, the risk circuits associated with predictive ROI-1 interconnect the dlPFC with the vmPFC, PCC/precuneus, visual and motor cortex, thalamus and fusiform face area (Fig. 2C). Critically, these regions largely overlap with the well-established DMN, characterized by higher activity during internally oriented thoughts and suppressed during performance of cognitively demanding tasks.^64^ As suppression of DMN activity is thought to be required for optimal performance of goal directed behaviour^62^; stronger DMN functional connectivity with this dlPFC locus may underlie the enhanced drug craving and impaired goal-directed behaviour seen in SUD individuals and may reflect less capacity to disengage DMN and in turn, enhance ECN to promote optimal performance, as suggested by large-scale network models of neuropsychiatric diseases.^55^^,^^62^ Acute and chronic stress and craving are known risk factors for SUD relapse.^65^^,^^66^ For example, stress induced alcohol craving correlates with increased vmPFC and PCC/precuneus activity, which can serve as a predictor of both shorter time to relapse and heavy drinking in alcohol use disorder patients.^66^ This risk circuits did not include any striatal regions, which are nominally related to processing reward and cue-induced craving mechanisms.^67^ That said, the current design focused on the relationship between the dlPFC functional connectivity and treatment outcome, whereas these striatal regions are more involved in the binge and intoxication stage of the addiction cycle,^1^ and are not necessarily expected to appear in the dlPFC-derived functional circuits that relate to relapse. However, such dlPFC interactions with the striatum may still exist in a more indirect way. For example, the DLPFC may interact with striatum but through its connectivity with the vmPFC found in the risk circuit.

This identified risk circuit of predictive ROI-1 is also termed the ‘β-network’ in a neuroeconomic based decision-making context and is thought to serve as a ‘drive’ network that primarily mediates immediate/short-term reward.^14^^,^^68^ Our results are consistent with this model by relating stronger functional connectivity between the dlPFC and these β-network regions to higher relapse likelihood. Given that steep discounting for delayed reward and the dysregulated interaction between these two networks is a known characteristic of SUD,^5^^,^^16^ that this ROI and its related circuits predicted relapse with a high level of accuracy is supportive of this framework. Enhanced β-network functional connectivity and/or blunted δ-network functional connectivity^69^ has also been associated with other behaviours dysregulated in SUD, including impulsivity and compulsive drug taking.^69^^,^^70^ Importantly, the protective and risk related dlPFC circuits identified herein also suggest that the *balance* of distinct dlPFC-based circuits related to immediate desires versus long-term planning is an important factor in individual treatment outcomes.

### The protective and risk circuits from predictive ROI-2 and ROI-3

The other two predictive dlPFC ROIs (ROI-2, Fig. 3A; and ROI-3, Fig. 4A) are located on the right hemisphere. The protective circuits associated with ROI-2 mainly include the bilateral dlPFC, IFG, right IPL, dmPFC, and left vlPFC, while the protective circuits associated with ROI-3 mainly consist of the bilateral dlPFC and dmPFC. While the vlPFC is typically associated with response inhibition,^18^^,^^71^ the dmPFC is crucially involved in social-emotional processes such as negative emotions and social judgments.^72^^,^^73^ It also plays an important role in stress-induced reinstatement of drug-seeking behaviour.^74^ Negative emotional states caused by drug withdrawal are a key causation of addiction relapse^28^ as well as one of the major causes of SUD establishment and maintenance.^3^

Relatedly, the dmPFC has been implicated in Theory of Mind (ToM),^75^ defined as the ability to attribute mental states to oneself and especially to others,^76^ a crucial feature in everyday life and social interaction that is impaired in SUD.^77^ It is well documented that cocaine dependent individuals show deficits in social-emotional processing.^46^^,^^78^ Our finding that the functional connectivity between dlPFC and dmPFC and vlPFC conveys protection against relapse may speak to the importance of the ability of the ECN, and dlPFC in particular, to regulate negative emotions and broader social-emotional processing necessary for successful outcomes in cocaine use disorder treatment. The inclusion of the IFG, IPL and the contralateral dlPFC in this protective circuit once again highlights the importance of a well-connected ECN in maintaining successful abstinence.

In contrast, the risk circuit associated with ROI-2 comprised primarily bilateral precuneus and the amygdala/hippocampus, while the risk circuit associated with ROI-3 included the bilateral cuneus/visual cortex, vmOFC, thalamus, fusiform gyrus and left precuneus. SUD has been characterized as a disease of dysregulated learning and memory, whereby maladaptive long-term memory formation pathologically usurps normal homeostatic processes to support continued drug use.^8^ Reward-related learning involves multiple memory systems, including reinforcement learning via the mesocorticolimbic pathway, and declarative memory systems of the medial temporal lobes (MTLs) and the precuneus.^79–81^ The mesocorticolimbic dopaminergic system, hippocampus and amygdala are progressively recruited in different phases of learning.^82^^,^^83^

SUD is also characterized by intense drug cravings that lead to stressful, aversive feelings, which may also lead to negative reinforcement, drug taking and relapse.^83^^,^^84^ Our finding that the functional connectivity between DLFPC and the medial temporal lobe–precuneus declarative memory system, along with the circuit between dlPFC and the mesocorticolimbic system (thalamus and vmOFC) may serve as risk factors to relapse is likely a reflection of the negative impact of both long-term alterations in declarative memory systems and the aberrant conventional reward-based, non-declarative memory systems dependent on ECN control of behaviour in SUD.

The identification of distinct risk and protective circuits from the three predictive ROIs also speaks to the heterogeneous phenotype of the disorder and may help characterize the well-known individual differences in cocaine dependence. For example, some cocaine dependent individuals may have a very strong drive coupled with relatively normal control ability while others may have moderate drive levels but impaired cognitive control. For others, ability to manage social and emotional challenges may contribute more strongly to their substance dependence than executive function, per se. Such hypothetical distinct endophenotypes are otherwise difficult to distinguish during standard pre-treatment assessments. Identifying individual cocaine use disorder subtypes based on circuits related to relapse risk vs. protection may allow assessment of which circuits are most impaired in a given individual, leading to more individualized and hopefully more efficacious interventions.

### Implications for neuromodulation-based treatment

Taken together, the three treatment outcome predictive dlPFC ROIs may have important implications for future TMS neuromodulation interventions. The predictive ROI-1 that showed high relapse predictive validity (AUC of 0.839) along with its functional circuits (both protective- and risk-circuit) is located within the left hemisphere dlPFC. Intriguingly, an open label pilot study in which rTMS significantly reduced cocaine relapse compared to a group receiving pharmacological interventions^40^ employed a left dlPFC stimulation target that is only 2.828 mm proximal (2 mm lateral and 2 mm superior) to this ROI. While providing a proof of concept, the consistency between our predictive validity and a real world TMS treatment outcome would suggest that targeting this locus with TMS could enhance the strength of the protective circuit and/or reduce the risk circuit strength, re-regulating the functional circuits towards configurations associated with longer abstinence. In contrast, the predictive ROI-2 and -3 are on the right hemisphere dlPFC. Although the majority of published neuromodulation studies have targeted the left dlPFC, there are reported anti-craving effects from right hemisphere dlPFC rTMS in various SUDs, including cocaine and alcohol.^85^^,^^86^

Previously, Fox et al.^87^ have explored the relationship between several dlPFC sub-regions and TMS treatment outcome in major depressive disorder patients and found that functional connectivity from various dlPFC TMS target sites was associated with different levels of depression treatment success. The wide range of treatment outcomes from targets all considered being ‘dlPFC’ illustrates the importance of appropriate target selection.

The present results expand our understanding of the role of dlPFC circuits in cocaine dependence. Previous studies employing on-line TMS during fMRI acquisition have demonstrated that following single-pulse dlPFC stimulation (although different target locating strategy), downstream brain regions of dlPFC are activated including the ACC, caudate, and vlPFC.^88^^,^^89^ Functional connectivity between networks were also modulated by TMS targeting corresponding network core regions using a paired-pulse paradigm.^90^ More recently, continuous theta-burst stimulation (cTBS) to the dlPFC was found to induce alterations in functional connectivity from the OFC, a brain region that is functionally and anatomically connected to dlPFC, but only indirectly reached by TMS.^91^ Given these acute effects of TMS on fMRI manifestations of brain activity, along with the consideration of state-dependency of TMS treatment^92^ in which baseline activity level of stimulated target/circuits can be manipulated by administering treatment related tasks during/before the TMS, it may be possible to enhance the functional connectivity of the protective circuits while maintain/reduce the functional connectivity of risk circuits, which may be usefully applied in future therapeutic neuromodulation interventions. Moreover, the strength of these circuits, and how they change over the course of a TMS treatment regime, may potentially serve as a prospective biomarker for treatment efficacy.

Notably, the cumulative statistically predictive influence of these three functional dlPFC regional circuits suggest that the variance in cocaine relapse explained by these circuits are at least partially distinct, with the left dlPFC ROI-1 likely primarily related to the executive functioning domain and the two right hemisphere dlPFC ROIs to social–emotional and memory domains. These data driven findings independently corroborate the model of multiple networks/circuits disruptions underlying SUD.^93^ Our findings further suggest that treatment efficacy might be enhanced from a multi-site stimulation design that includes more than one of the identified functional circuits.

Finally, our current analysis focused on the relationship between functional circuits of the dlPFC and cocaine relapse. Other functional circuits have previously been identified that predict cocaine relapse (some were identified using this same dataset), e.g. between the PCC and the hippocampus,^44^ between the temporal pole and the mPFC,^46^ as well as from large-scale networks such as the ECN.^45^^,^^94^ However, the current analysis was specifically focused on the dlPFC, motivated by its prominent potential and feasibility as a neuromodulation target, and incorporated contemporary statistical rigour which allowed us to identify two functionally distinct circuits, i.e., a protective and risk circuit, for each of the three predictive ROIs. It is worth noting that each pair of circuits are functionally connected to the same dlPFC ROI, and thus care should be taken if utilizing these predictive ROIs as neuromodulation targets since different individuals might not respond to a given TMS intervention in the same way. This may also explain the variation in treatment outcome seen in neuromodulation treatment studies (see Table 1 in Hanlon et al.^95^). Future studies should prospectively test the effects of TMS intervention targeting these functional circuits, on an individual basis, to examine how they change across the treatment regimen and their relationship with treatment outcome efficacy.

### Limitations

Despite the novelty and potential treatment implications of our study, several limitations should be considered. Our sample included only five females and future work should address the possibility of gender differences. We did not include alcohol consumption as a covariate in our prediction model despite its impact on brain structure and function and its well-known comorbidity with cocaine dependence^27^^,^^96^ because this information was originally acquired as an ordinal rather than a continuous variable. However, our participants reported on average consuming less than 2 alcohol drinks in the previous two weeks (based on the ordinal variable from 0 to 6), suggesting a low alcohol influence in the present cohort. Clinically, factors such as ‘previous relapse episodes’ and ‘treatment-seeking motivation’ might be relevant to relapse as well. However, as this was a follow-up study, we were not in a position to retrospectively acquire these data. Therefore, the functional circuits we observed herein are related to the overall likelihood of relapse in the follow-up period and cannot reveal the relationship to an individual’s previous relapse episodes or treatment-seeking motivation. Finally, our data were measured after psychosocial treatment, and therefore cannot speak to whether the co-variance between these functional circuits and the follow-up relapse, were pre-existing or a consequence of the treatment. However, we were not attempting to tease apart the role of the specific intervention per se, but rather to determine whether functional connectivity after treatment can be used to predict subsequent relapse. That said, the results do illustrate an important relationship to successful abstinence.

## Conclusions

We built Cox-regression based prediction models using the functional connectivity from 98 surface loci covering the entire dlPFC bilaterally and identified three loci with high prediction power for cocaine relapse, one on the left hemisphere, which is practically the same locus that used as TMS target in a previous open label study that significantly reduced cocaine relapse, and two on the right hemisphere. These ROIs may serve as potential targets to be tested for neuromodulation treatment efficacy for addiction. We also identified three sets of functionally distinct dlPFC-based circuits related to these ROIs that conveyed protection against and risk for cocaine relapse. These findings support important roles for ‘bottom up’ drive to use drug and ‘top down’ cognitive control over behavioural choice, emotional and social functioning, and dysregulated memory and learning related circuits, consistent with contemporary models of SUD.^3^^,^^93^ Future studies need to assess the ability of TMS to alter these circuits in therapeutically useful ways. Finally, these data highlight the importance of the balance between protective and risk factors in determining treatment outcome and the potential utility of resting-state functional connectivity as a clinically relevant biomarker to guide individualized therapy for drug dependence.

## Supplementary material

Supplementary material is available at *Brain Communications* online.

## Supplementary Material

fcab120_Supplementary_Data

## Abbreviations

ACC =: anterior cingulate cortex
AUC =: area-under-curve
BOLD =: blood-oxygen-level-dependent
dlPFC =: dorsolateral prefrontal cortex
DMN =: default mode network
dmPFC =: dorsomedial prefrontal cortex
DSM =: the Diagnostic and Statistical Manual of Mental Disorders
ECN =: executive control network
FFA =: fusiform face area
fMRI =: functional MRI
HR =: relative hazard ratio
IFG =: inferior frontal gyrus
IPL =: inferior parietal lobule
IQ =: intelligence quotient
mPFC =: medial prefrontal cortex
PCC =: posterior cingulate cortex
ROC =: receiver operating characteristic
ROI =: region-of-interest
rTMS =: repetitive TMS
SUD =: substance use disorders
TMS =: transcranial magnetic stimulation
ToM =: theory of mind
TR =: repetition time
UDS =: urine drug screen
vlPFC =: ventrolateral prefrontal cortex
vmOFC =: ventromedial orbitofrontal cortex
vmPFC =: ventromedial prefrontal cortex
