# Supplementary material for: Functional connectivity of dorsolateral prefrontal cortex predicts cocaine relapse: implications for neuromodulation treatment
Source: Brain Commun. 2021 Jun 2;3(2):fcab120. doi: 10.1093/braincomms/fcab120 (PMC8226190; doi:10.1093/braincomms/fcab120)
Supplement: fcab120_Supplementary_Data [file fcab120_supplementary_data.pdf]

Supplementary material for

Functional connectivity of dorsolateral prefrontal cortex predicts cocaine relapse: Implications for neuromodulation treatment

Tianye Zhai; Betty Jo Salmeron; Hong Gu; Bryon Adinoff; Elliot A. Stein; Yihong Yang

Content list:

Supplementary Fig. 1. Schematic diagram of analytical pipeline

Supplementary Table 1. Brain regions in the group level heat map of protective circuit and risk circuit for predictive ROI-1, predictive ROI-2, and predictive ROI-3

Supplementary Table 2. Cocaine relapse prediction validity of prediction models built using six control seed ROIs

Supplementary Table 3. Relationship between the behavioral/psychological measurements and the functional circuits of predictive- ROI-1, ROI-2, and ROI-3

Supplementary Table 4. Cocaine relapse prediction validity of the behavioral/psychological measurements

Supplementary Fig. 1. Schematic diagram of analytical pipeline

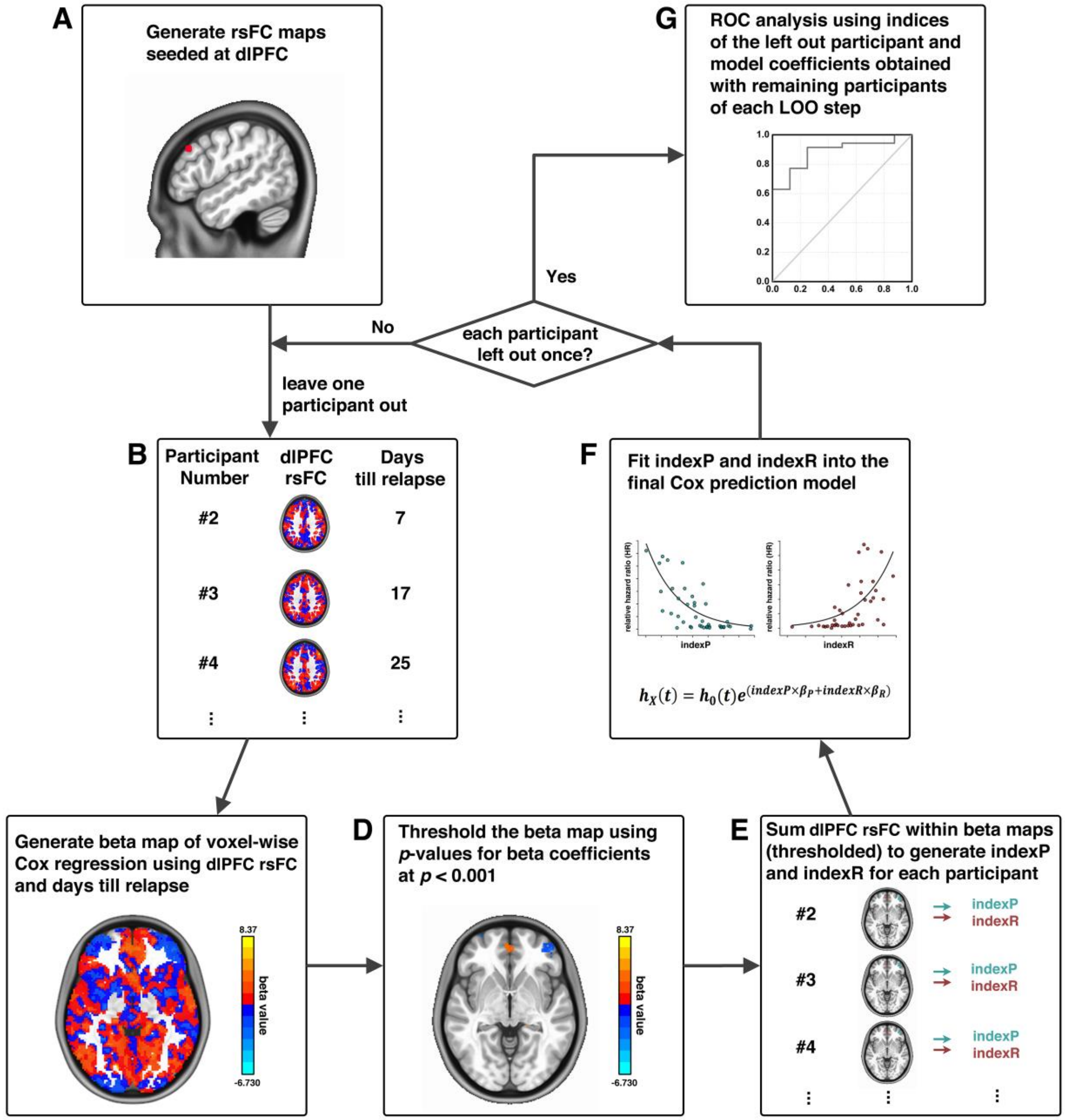

The analytical pipeline from one exemplar dlPFC locus. A dlPFC ROI is selected as a seed (A); the whole-brain rsFC of this dlPFC seed is calculated for each participant (B); a voxel-wise Cox regression is conducted using dlPFC rsFC and days until relapse to generate beta maps (C); beta maps of Cox regression is thresholded (D); generation of indexP and indexR by linearly summation of the dlPFC rsFC within the thresholded beta maps for the negative and positive beta voxels, respectively (E); construction of the final prediction model by fitting indexP and indexR into the Cox model (F); the steps from B to F were repeated in a leave-one-out (LOO)

manner, and after each participant is left out once, a final ROC analysis evaluates the prediction model (G).

Abbreviations: rsFC, resting-state functional connectivity; dlPFC, dorsolateral prefrontal cortex; ROI, region-of-interest; ROC, Receiver-Operating-Characteristics; LOO, leave-one-out

Supplementary Table 1. Brain regions in the group level heat map of protective circuit and risk circuit for predictive ROI-1, predictive ROI-2, and predictive ROI-3

Seed	Circuit	Brain Region	MNI Coordinates (LPI)			Cluster Size (mm^3)
			X	Y	Z	
Predictive ROI-1	Protective	L-IFG	-44	52	6	600
		L-IPL	-54	-42	54	160
		R-IFG	50	46	-12	112
		L-dlPFC	-38	36	38	112
		R-dlPFC	42	22	18	88
		L-dlPFC	-42	10	58	72
		L-ITG	-58	-36	-24	56
		L-Insula	-38	0	-2	56
		R-dlPFC	30	54	8	48
		R-IPL	50	-50	32	32
	Risk	L-Cuneus	-14	-78	38	1152
		R-Cuneus	20	-66	28	736
		R-FFA	36	-50	-18	632
		B-vmPFC	0	48	-6	336
		R-PostCG	62	-24	22	280
		B-vmPFC	2	44	-16	232
		R-mOFC	10	26	-26	168
		R-Cuneus	14	-82	46	120
		R-Fusiform	28	-68	-18	112
		R-PCC	2	-42	18	112
		L-Lingual	-18	-64	4	96
		L-Lingual	-20	-70	-10	88
		L-IOG	-32	-78	-10	80
		L-Precuneus	-14	-48	34	72
		R-Fusiform	36	-40	-14	64

		L-Insula	-32	24	8	64
		R-Fusiform	28	-82	-10	56
		B-SubCG	0	22	-12	40
		R-MTG	50	-42	10	32
		L-MOG	-32	-90	16	32
		R-Precuneus	8	-66	50	32
		L-Thalamus	-2	-10	6	24
Predictive ROI-2	Protective	R-dmPFC	6	50	38	128
		L-vlPFC	-16	36	-24	96
		L-dlPFC	-50	18	40	96
		R-dlPFC	34	46	24	88
		L-IFG	-50	42	-4	72
		L-dlPFC	-40	44	20	56
		L-IFG	-44	56	-10	40
		R-IFG	44	50	4	16
		R-IPL	44	-24	58	16
	Risk	B-Precuneus	-2	-50	62	2096
		L-MTG	-48	-70	2	176
		R-Fusiform	30	-60	-18	128
		R-PreCG	48	6	24	128
		L-SMA	-10	-8	64	120
		L-SMA	-4	-8	52	112
		R-MTG	44	-58	22	80
		L-Thalamus	-6	-12	14	64
		L-Hippo	-34	-22	-16	56
		R-Lingual	4	-70	2	56
		L-Precuneus	-4	-76	46	56
		L-Precuneus	-14	-42	46	56
		L-STG	-50	-36	16	48

		R-Amy	22	0	-20	40
		R-STG	56	-30	14	40
		R-MOG	50	-80	18	40
		L-Amy	-28	-4	-18	32
Predictive ROI-3	Protective	R-dlPFC	34	46	24	200
		L-dmPFC	-8	6	56	136
		R-dlPFC	46	42	20	72
		L-dlPFC	-26	50	28	48
		R-SFG	26	56	4	40
		R-Precuneus	14	-48	40	40
		R-dmPFC	4	44	44	24
	Risk	L-Precuneus	-16	-70	24	1208
		R-Precuneus	24	-62	20	760
		L-Cuneus	-8	-64	10	632
		R-Cuneus	10	-56	6	504
		L-MTG	-42	-88	18	448
		L-Fusiform	-30	-46	-20	440
		L-Precuneus	-4	-48	58	200
		L-Fusiform	26	-46	-20	176
		R-Fusiform	-24	-52	-4	136
		R-IFG	44	32	12	104
		R-MTG	46	-76	18	88
		R-SOG	24	-66	34	88
		R-mOFC	6	22	-28	64
		L-Fusiform	-30	-60	-10	56
		L-MFG	-24	28	-16	40
		L-Thalamus	-8	-28	10	32
		R-Parahippo	20	-18	-20	16

Abbreviations: IFG, inferior frontal gyrus; IPL, inferior parietal lobule; dlPFC, dorsolateral prefrontal cortex; ITG, inferior temporal gyrus; FFA, fusiform face area; vmPFC, ventromedial prefrontal cortex; Post CG, postcentral gyrus; mOFC, medial orbitofrontal cortex; PCC, posterior cingulate cortex; IOG, inferior occipital gyrus; SubCG, subcallosal gyrus; MTG, middle temporal gyrus; MOG, middle occipital gyrus; dmPFC, dorsomedial prefrontal cortex; vlPFC, ventrolateral prefrontal cortex; Pre CG, precentral gyrus; Hippo, hippocampus; STG, superior temporal gyrus; Amy, amygdala; SFG, superior frontal gyrus; SOG, superior occipital gyrus; MFG, middle frontal gyrus; Parahippo, parahippocampal gyrus.

Supplementary Table 2. Cocaine relapse prediction validity of prediction models built using six control seed ROIs

	L-Auditory	R-Auditory	L-Motor	R-Motor	L-Visual	R-Visual
AUC of the ROC curve	0.471	0.629	0.493	0.493	0.614	0.646
p-value	0.629	0.206	0.579	0.565	0.237	0.167

Abbreviations: ROI, region-of-interest; AUC, Area-Under-Curve; ROC, Receiver-Operating-Characteristics;

Supplementary Table 3. Relationship between the behavioral/psychological measurements and the functional circuits of predictive- ROI-1, ROI-2, and ROI-3

Measurements	Index	predictive ROI-1		predictive ROI-2		predictive ROI-3	
		indexP r (*p*-value)	indexR r (*p*-value)	indexP r (*p*-value)	indexR r (*p*-value)	indexR r (*p*-value)	indexR r (*p*-value)
BIS-11a (n=42)	Attention	0.133 (*p*=0.400)	-0.025 (*p*=0.874)	0.024 (*p*=0.880)	0.075 (*p*=0.638)	0.033 (*p*=0.838)	-0.059 (*p*=0.713)
	Motor	0.133 (*p*=0.402)	-0.151 (*p*=0.339)	0.094 (*p*=0.539)	0.049 (*p*=0.757)	0.219 (*p*=0.164)	-0.113 (*p*=0.475)
	Non-planning	0.195 (*p*=0.216)	-0.057 (*p*=0.722)	0.073 (*p*=0.648)	-0.261 (*p*=0.095)	0.267 (*p*=0.088)	-0.149 (*p*=0.346)
	Total Score	0.199 (*p*=0.207)	-0.114 (*p*=0.471)	0.090 (*p*=0.569)	-0.043 (*p*=0.788)	0.234 (*p*=0.136)	-0.142 (*p*=0.371)
CCQ (n=42)	Total Score	-0.014 (*p*=0.929)	-0.004 (*p*=0.983)	-0.087 (*p*=0.586)	0.149 (*p*=0.345)	-0.088 (*p*=0.581)	0.082 (*p*=0.606)
CPT (n=40)	Omission Error	0.054 (*p*=0.743)	-0.078 (*p*=0.634)	-0.012 (*p*=0.942)	-0.012 (*p*=0.941)	0.159 (*p*=0.327)	-0.007 (*p*=0.968)
	Commission Error	-0.034 (*p*=0.835)	-0.018 (*p*=0.911)	-0.063 (*p*=0.701)	0.091 (*p*=0.576)	0.025 (*p*=0.878)	0.140 (*p*=0.388)
	Reaction Time	0.107 (*p*=0.510)	-0.095 (*p*=0.561)	0.229 (*p*=0.155)	-0.233 (*p*=0.148)	0.183 (*p*=0.259)	-0.193 (*p*=0.234)
IGT (n=38)	Total Score	0.043 (*p*=0.798)	-0.056 (*p*=0.740)	0.228 (*p*=0.169)	0.115 (*p*=0.494)	0.067 (*p*=0.692)	-0.050 (*p*=0.769)
WCST (n=37)	Perseveration	-0.182 (*p*=0.281)	0.103 (*p*=0.546)	-0.070 (*p*=0.682)	0.196 (*p*=0.246)	-0.225 (*p*=0.181)	0.247 (*p*=0.141)

Abbreviations: ROI, region-of-interest; BIS, Barrett Impulsivity Scale (BIS-11a); CCQ, Cocaine Craving questionnaire; CPT, Conners' Continuous Performance Test II; IGT, Iowa Gambling Task; WCST, Wisconsin Card Sorting Task

Supplementary Table 4. Cocaine relapse prediction validity of the behavioral/psychological measurements

Measurements	Index	AUC of the ROC curve	*p*-value
BIS-11a (n=42)	Attention	0.574	0.352
	Motor	0.563	0.388
	Non-planning	0.596	0.318
	Total Score	0.585	0.340
CCQ (n=42)	Total Score	0.625	0.233
CPT (n=40)	Omission Error	0.617	0.241
	Commission Error	0.629	0.222
	Reaction Time	0.668	0.156
IGT (n=38)	Total Score	0.751	0.052
WCST (n=37)	Perseveration	0.624	0.242

Abbreviations: ROI, region-of-interest; AUC, Area-Under-Curve; ROC, Receiver-Operating-Characteristics; BIS, Barrett Impulsivity Scale (BIS-11a); CCQ, Cocaine Craving questionnaire; CPT, Conners' Continuous Performance Test II; IGT, Iowa Gambling Task; WCST, Wisconsin Card Sorting Task
